# Cytogenetics Findings in a Histiocytic Sarcoma Case

**DOI:** 10.1155/2012/428279

**Published:** 2012-06-03

**Authors:** J. M. Alonso-Dominguez, M. Calbacho, M. Talavera, C. Villalon, L. Abalo, J. V. Garcia-Gutierrez, S. Lozano, M. Tenorio, J. Villarrubia, J. Lopez-Jimenez, M. T. Ferro

**Affiliations:** ^1^Servicio de Hematología, Hospital Ramón y Cajal, Carretera de Colmenar Km 9, 100 28049 Madrid, Spain; ^2^Servicio de Genética, Hospital Ramón y Cajal, Carretera Colmenar KM 9, 100 28034 Madrid, Spain

## Abstract

Histiocytic sarcoma (HS) is a neoplasm derived from histiocytes. Its diagnosis was not clear until its immunohistochemistry profile was correctly established. Not much is known about its genetic properties. We report a case of a 48-year-old male patient whose bone marrow was almost completely occupied by monomorphic medium size neoplastic cellularity. Its immunohistochemical profile was CD68^+^, CD4^+^, CD45^+^ with negativity of other dendritic cells, and other lineage markers. Cytogenetic study showed 4 related clones: one with trisomy 8 and extra material on the short arms of chromosome 4; a second line with tetrasomy of chromosome 8, add(4)(p16); the third clone had the same alterations as the previous and deletion of chromosome 3 at q11; the fourth line had tetrasomy 8 and translocation t(3;5)(q25;q35). To our knowledge this is the first HS case showing chromosome 8 trisomy and tetrasomy and the other described alterations.

## 1. Introduction

Histiocytic sarcoma (HS) is a neoplasm derived from histiocytes, also called macrophages. These cells are derived from bone marrow monocytes that migrate from peripheral blood to different tissues, although local proliferation also exists [1]. Therefore, it is a myeloid-derived neoplasm.

Its diagnosis was not clear until its immunohistochemical profile was correctly established. There is expression of at least one histiocytic marker: CD 68, CD 163, or lysozyme with negativity of Langerhans cell (CD1a, langerin) and follicular dendritic cell (CD21, CD35) markers. In addition CD4, CD45, CD45RO, and HLA-DR are normally positive. Epithelial membrane, melanoma, B-cell, T-cell, and myeloid markers are also negative [2–4]. There are few *bona fide* cases reported given that many of the neoplasms thought to be HS were T-lineage-associated hematolymphoid neoplasms [5]. Some cases occur in patients with mediastinal germ cell tumours due to the presence in these tumours of multipotential cells capable of giving rise to colonies containing macrophages and other cell types [6]. Histiocytic sarcomas usually lack clonal immunoglobulin heavy chain (IGH) or T-cell receptor (TCR) gene rearrangement, although some cases have been reported that show these clonality features [7–9].

Little is known about the cytogenetic features of this neoplasm. A case reported showed a clonal cytogenetic abnormality including t(14;18) which was confirmed by fluorescence in situ hybridization. It also had clonal IGH gene rearrangement [9]. Other authors described a HS derived from a chronic myelomonocytic leukemia (CMMoL). HS cells were all tetraploid and had octasomy to decasomy 8 and the primitive CMMoL cells were hyperdiploid with an extra chromosome 8 [10]. We report a HS case with multiple gains of chromosome 8 and other additional abnormalities.

## 2. Case Report

**Figure 1:**
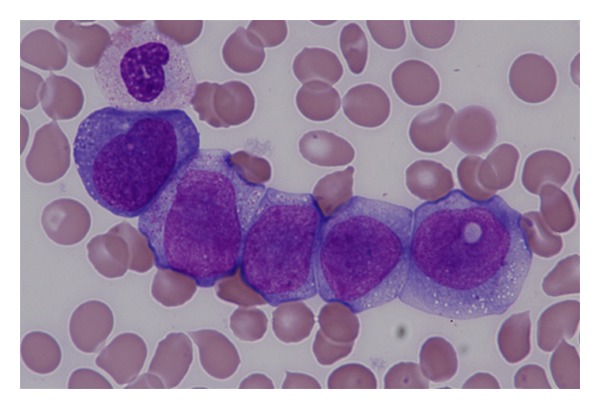
Peripheral blood slide review.

A 48-year-old male patient, without interesting medical records but a congenital pendular nystagmus, was admitted to hospital with pancytopenia and pain in right shoulder and pelvis. On a slide review, 4% of blasts (percentage referred to total nucleated cells) were observed and 12% of erythroblasts. The blastic cells were monomorphic, medium size, with nuclei of variable shape, round or split, with nucleoli, and presence of vacuoles, often large (Figure 1).

A bone marrow biopsy was carried out. Bone marrow was occupied almost completely by the same cells found in peripheral blood. Immunohistochemically CD68, CD4 and CD45 were positive and dendritic cells markers (CD1a, S100 and CD23) were negative excluding neoplasm derived from these related cells. Epithelial (CKAE1/AE3, CAM52), melanoma (HMB45), lymphoid (CD20, CD3, CD30, TdT), myeloid (MPO), and blast markers (CD34) were also negative. 

Flow cytometry identified 42% of cells with the following profile: CD33+, CD45+, CD14−, CD11b−, CD34−, CD64+, DR+, CD4+, CD56+, CD13±.

Body Scan computed tomography (CT) showed splenomegaly of 16.5 cm and adenopathies of 2.5 cm in hepatic hilus and celiac trunk. A hypodensity surrounding the portal radicle that was attributed to extramedullar haematopoiesis was also detected. This could explain the splenomegaly observed.

The patient was treated with CHOP ×4. In the haematological cytology of the bone marrow carried out following the third chemotherapy cycle, blasts had decreased to 40% of the total cellularity. 

One month after the last cycle, he was readmitted due to a paralysis of VI cranial nerve and paraesthesia in the jaw region. Minimal occupancy of sphenoidal sinus was observed in CT. Three lumbar punctures were negative for neoplastic cell. Cytarabine, methotrexate and dexamethasone were injected with each puncture. In the bone marrow aspirate the percentage of blasts rose to 99%.

**Figure 2:**
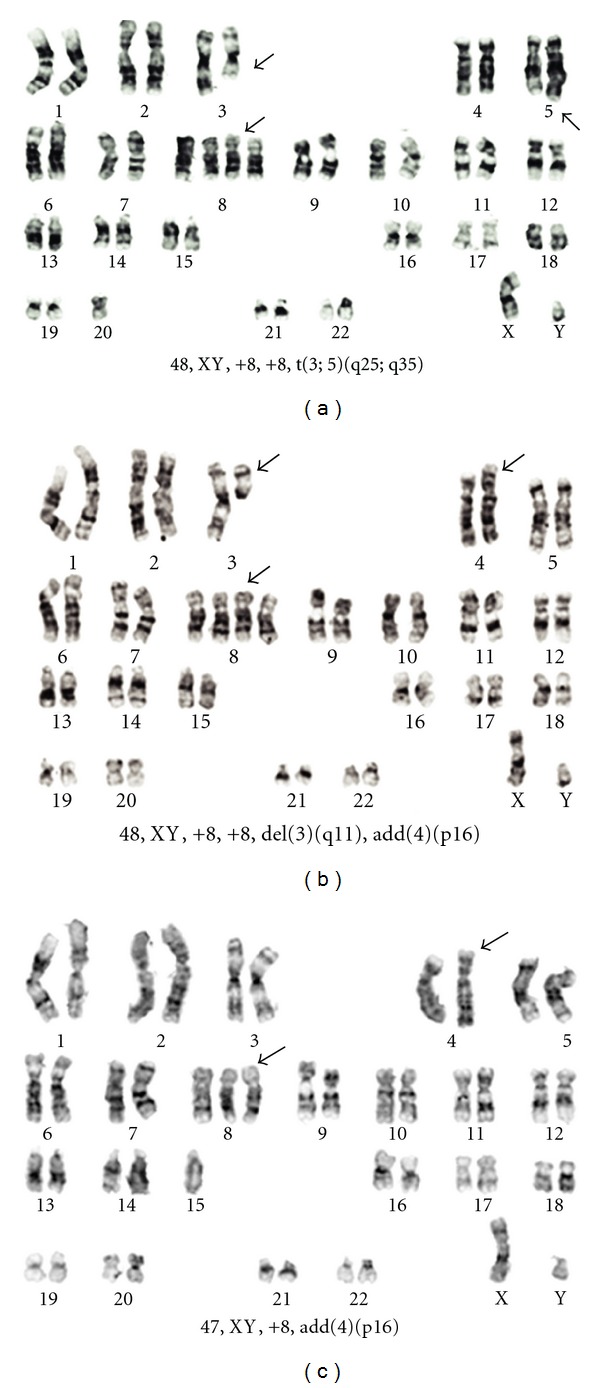
Histiocytic sarcoma karyotype. Alterations not pointed by arrows were not clonal.

Cytogenetic study was performed on bone marrow 24 hours culture and showed 4 related clones: one with trisomy 8 and extra material on the short arms of chromosome 4; a second line with tetrasomy of chromosome 8, add(4)(p16); the third clone had the same alterations as the previous and deletion of chromosome 3 at q11 and the fourth line had tetrasomy 8 and translocation t(3;5)(q25;q35), similar to that described in myeloid disorders [11] (Figure 2).

The karyotype was:

*47, XY, add(4)(p16), +8[**2]/48, XY, add (4)(p16), +8, +8[**2]/48, XY, del(3)(q11), add(4)(p16), +8, +8[**2]/48, XY, t(3;5)(q25;q35), +8, +8[**5]/46, XY[**2].*

Nonclonal abnormalities were found. One of them with pentasomy 8 and other with del(7)(p11) had also a tetrasomy 8, del(3)(q11), and add(4)(p16). This last cell might be derived from the third clone described. 

With FISH analysis, there were disomic, trisomic, and tetrasomic cells for chromosome 8. The FISH study using p53, MLL, and IgH probes were also normal.

Two other different chemotherapy cycles containing ifosfamide, carboplatin plus etoposide, and methotrexate plus cytarabine were administered. The patient died of progressive disease 6 months after diagnosis.

## 3. Discussion

Our case was studied by using different techniques and its immunohistochemical pattern was conclusive in order to establish the correct diagnosis following current diagnosis criteria [4].

To our knowledge this is the first HS case showing chromosome 8 trisomy and tetrasomy and the other described alterations. The most likely evolution of the neoplasm was from an initial trisomy 8 clone that gained an extra chromosome 8 copy and developed the additional alterations. 

Trisomy 8 is one of the most common chromosomal abnormalities in myeloid malignancies occurring in approximately 10–20% of cytogenetically abnormal acute myeloid leukemias (AML), myelodysplastic syndromes (MDSs), and myeloproliferative disorders (MPD) [12–14]. 

Chromosome 8 gains, from trisomies to pentasomies, have been described in a series of myeloid malignancies corresponding predominantly to neoplasm with monocytic differentiation (AML-M4, AML-M5, and CMMoL) [14–20].

Taking this into consideration, our cytogenetic findings in this HS case support its myelomonocytic origin and shed light on genetic alterations underlying this uncommon neoplasm.

HS is a very aggressive tumour according to the few series published so far [3, 21]. The extension of the disease and the size seem to be the most important prognostic factors. Our case had bone marrow involvement. This advanced stage and the complex karyotype could explain the severity of this case.
